# Computational fluid dynamics investigation on the irrigation of a real root canal with a side-vented needle

**DOI:** 10.1186/s12903-024-03966-8

**Published:** 2024-03-09

**Authors:** Mingzhou Yu, Yi Li, Mengdie Zhao, Zhengqiu Huang, Na Zhou, Hanhui Jin

**Affiliations:** 1 School of Aeronautics and Astronautics, Zhejiang University, Hangzhou, 310027 People’s Republic of China; 2 Aerosol Science and Technology Laboratory, China Jiliang University, Hangzhou, 310018 People’s Republic of China; 3 Stomatology Hospital, School of Stomatology, Zhejiang University School of Medicine, Clinical Research Center for Oral Diseases of Zhejiang Province, Key Laboratory of Oral Biomedical Research of Zhejiang Province, Cancer Center of Zhejiang University, Hangzhou, 310006 People’s Republic of China; 4 State Key Laboratory of Clean Energy Utilization, Zhejiang University, Hangzhou, 310027 People’s Republic of China

**Keywords:** Root canal curvature, Needle working length, Side-vented needle, Computational fluid dynamics, Effective irrigation

## Abstract

**Background:**

Root canal therapy is one of the main treatments for root canal diseases, and effective irrigation is the key to successful treatment. Side-vented needle is one of the commonly used needle types in clinic. In the real root canal, due to the influence of the curvature of the root canal, the irrigation flow field in different needle directions shows obvious differences. At the same time, changes in root canal curvature and working depth will lead to changes in irrigation efficiency and the flow field. Both the mainstream of the irrigation flow and the shear stress near the wall changes significant. Consequently, either the replacement in the root canal or the removal efficiency of the smear layers is apparently modified.

**Materials and methods:**

In this paper, the permanent root canal of the maxillary first molar prepared until 15/04 were scanned by micro-CT, and then imported into the software for 3D reconstruction. The key parameters of flushing efficiency of 30G side needle at different working depths of 4.75 mm, 5 mm, 5.25 mm and 5.5 mm were compared. Meanwhile, the simulated models with different curvatures of 0°, 5°, 10°, 20° and 30° based on the real root canal were reconstructed to investigate the curvature effect on the irrigation efficiency.

**Results:**

The results show that moderate working depth (such as 4.75 mm and 5.25 mm in present paper) helps to improve the replacement capacity of irrigation flow. At the same time, the apical pressure decreased as the working depth increased. The curvature of the root canal seriously affects the removal depth of the smear layers of the root canal. A root canal with a large curvature (especially 20° and 30°) can significantly improve the difficulty of irrigation.

**Conclusions:**

(1) Moderate working depth helps to improve the displacement capacity, the ERD of the irrigation flow is generally improved at the working depths of 4.75 mm and 5.25 mm, and the apical pressure will decrease with the increase of working depth. (2) The large curvature of the root canal can significantly improve the difficulty of irrigation. The curvature of the root canal can severely influence the removal depth of the smear layer on the wall. It can be found both the span and the depth of the ESS for little curvatures (5° and 10°) root canals are higher than those for large curvatures (20° and 30°).

## Introduction

Root canal irrigation is an important step in root canal preparation procedures and has a great impact on the success rate of root canal treatment. Its aim is the removal of the residue and smear layer with necrotic tissues, debris and bacteria in the root canal, and to clean or disinfect the root canal surface where the mechanical instruments can not reach [1–3].

Together with the chemical and physical properties of the irrigation, the flow field inside the root canal basically determines the efficiency of root canal irrigation. Because of the limitations of experimental observation and measurement, the Computational Fluid Dynamics (CFD) method is widely used research into root canal irrigation, including the influence of needle type [4], irrigation flow velocity [5], root canal size [6] and irrigation fluid temperatures [7]. The models used in most of these studies are of simple artificial tapered root canals [5, 8], whose shape differs from that of actual human root canals in curve, convex, concave and wall abnormality [9, 10]. The influence of the irregular shape of real teeth is commonly neglected, leading to the over-or-underestimation of irrigation efficiency [11],especially when the shape of an actual root canal is coupled with different types of irrigation needle. Root canal irrigation studies based on real root canals yield valid conclusions and reliable clinical references [12]. To date, very few CFD studies have been done based on real human root canals. These studies discuss the influence of positive and negative pressure irrigation systems, irrigation fluid flow rate [11] and the position of the side needle on irrigation effectiveness [13] for a specific root canal. The curvature of the root canal, a cardinal shape parameter, has rarely been investigated using CFD method.

Because of the complex shape of real root canals, an appropriate type of needle can greatly enhance cleaning. To evaluate different types of needles in root canal irrigation is vital. In our earlier numerical investigations, the irrigation efficiency of a flat needle was studied at different working depths and canal curvatures. Although side-vented needles, which clean effectively and have low risk of apical extrusion, have had wide acceptance [14, 15], their efficiency in actual root canal irrigation hasn’t been assessed systematically. Compared to the common flat needle, the cleaning efficiency of a side-vented needle can be improved by adjusting the orientation of the needle’s aperture during irrigation despite the irregular shape of the root canal [13]. Because of the orientation of the irrigation aperture and irregular shape of a real root canal, evaluating the effectiveness of a side-vented needle versus a flat needle is complex.

In this study, the prepared human maxillary root canal model was obtained using micro-computer tomography (micro-CT) technology [16]. CFD was used to evaluate the effects of root canal irrigation with different working depths and different pore sizes on the actual root canal [17]. At the same time, the irrigation efficiency of the side-vented needle for different root canal curvatures was investigated for various aperture orientations.

## Materials and methods

### Model reconstruction

The study was approved by the Ethics Committee of the Affiliated Hospital of Stomatology (certificate 2018–030). Extracted maxillary first molars with complete roots and mature apices were obtained. After removal of dental remnants and soft tissue from the root surface, the teeth were stored in 0.1% thyme solution at 4 °C until use. A tooth with a separate palate root and a curvature of 23.4°was selected by micro-CT scanning (Scanco-Medical micro-CT 100 system; Scanco Medical, Bassersdorf, Switzerland). The pulp chamber was routinely accessed, a 10 K-file was inserted into the palate canal to establish apical patency, the working length (WL) was determined by passing the 10 K-file through the apical foramen and withdrawn 1 mm.

The palate canal was instrumented and shaped using a size 15/04 Profile Vortex Blue file (Dentsply Tulsa Dental Specialties, Tulsa, OK) up to the WL. After placing the patency file, before and after using the 15/04 Profile Vortex Blue file, the root canal was irrigated with 5% NaOCl. The root canal was dried using the moisture-absorbent paper tip, and the root canal was scanned again with the Micro-CT system. A total of 685 cross-sectional slice images (TIFF format) with a pixel size of 30 mm in each direction were obtained from the CT system. The preliminary reconstructed root canal model was obtained after pixel threshold division and model reconstruction of Mimics. After smoothing and optimizing the preliminary root canal model in Geomagic, the real human root canal model was obtained in standard template library (STL) format.

**Fig. 1:**
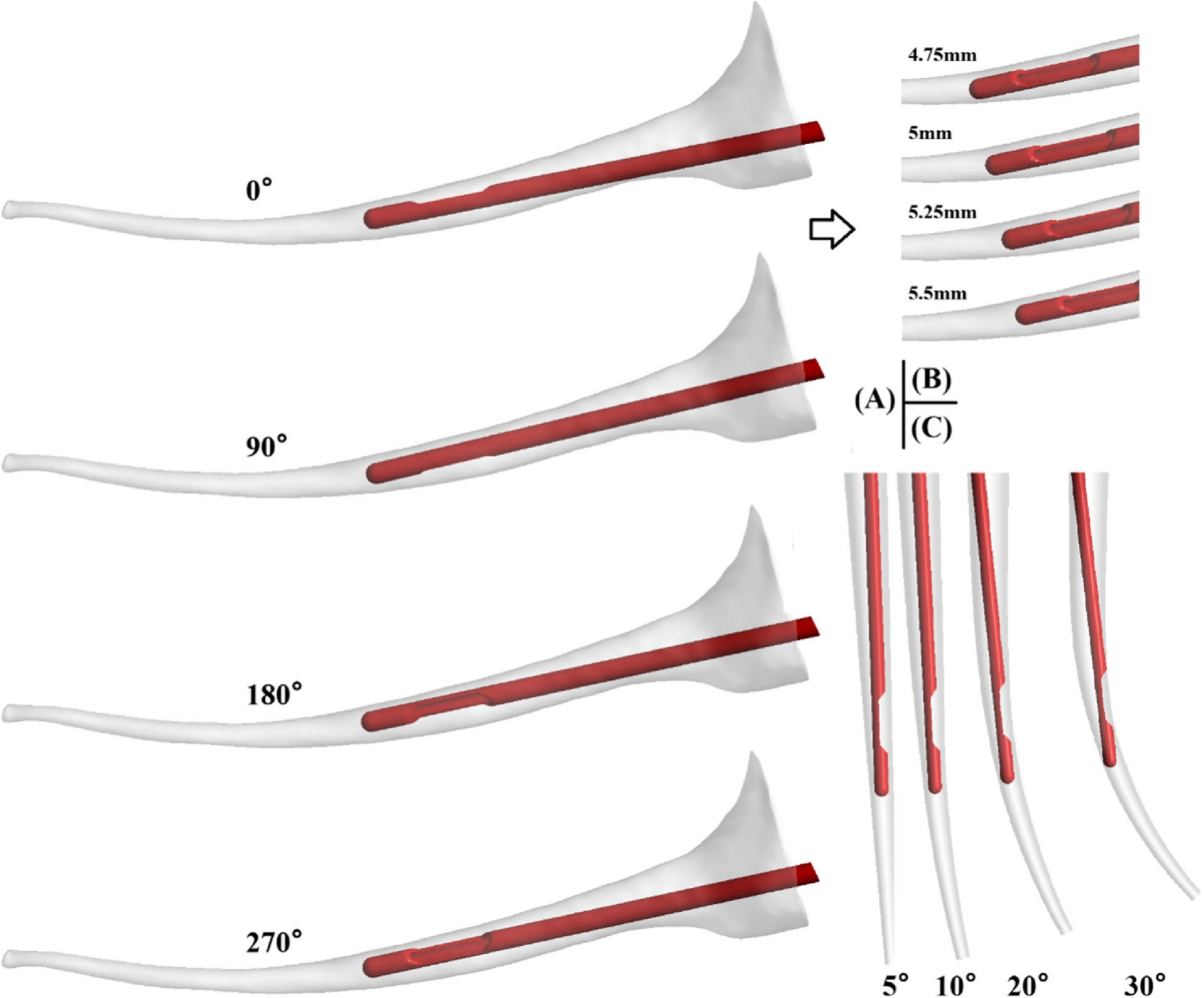
Geometric model of root canal irrigation; (**A**) Different orientation of the side-vented needle, (**B**) Different working depths, (**C**) Different orientations of the side-vented needle in the root canals with differing curvatures

A 30G side-vented needle was adopted [18, 19], having external and internal diameters D_ext_ = 320 μm, D_int_ = 196 μm, and length L = 31 mm. The numerical simulation flow field for the real root canal model was obtained by combining the needle model and the real root canal reconstruction model. The single aperture of the side-vented needle was rotated to four different angles (0°,90°,180°,270°) to explore the overall irrigation effect of the side-vented needle in the irregular real root canal (as shown in Fig. 1(A)). At the same time, four different working depths were modeled and simulated to evaluate their influence on the cleaning flow field, namely 4.75 mm, 5 mm, 5.25 mm, 5.5 mm (as shown in Fig. 1(B)). In addition, 911,204, 923,256, 911,126, and 914,843 computational unstructured tetrahedral meshes were arranged to conduct CFD simulation.

In order to explore the irrigation effect of the side-vented needle in root canals having different curvature, this study changed the curvature while keeping other parameters constant. Form the root canal curvature range, 5°, 10°, 20° and 30°curvatures were selected for comparison (as shown in Fig. 1(C)). With each curvature, the needle was circumferentially fixed at 0°, 90° 180° and 270°, respectively, to investigate the influence of aperture orientation.

### Boundary conditions and numerical simulation

5.25% sodium hypochlorite solution was adopted as the irrigation, with density of 1.04 g/cm^3^ and viscosity of 1.3 × 10^−3^ Pa*s [20]. The surfaces of the needle and root canal were regarded as rigid, impermeable walls, and a non-slip boundary condition was adopted in the simulation. The k-ω SST turbulence model that had been validated in our earlier studies was used [11, 21]. Similar to the commonly used clinical irrigation flow of 0.26 mL/s [5, 22], an inflow velocity of 8.6 m/s was adopted so that the effect of different working depths and root canal curvatures could readily be evaluated. Numerical simulation was carried out with software Ansys Fluent 18.1 (ANSYS Inc., Canonsburg, PA, USA).

## Results

The irrigation process of the side-vented needle in a real root canal can represent clinical irrigation. This study evaluated the influence of working depth (4.75 mm, 5 mm, 5.25 mm, 5.5 mm) and root canal curvature (5°, 10°, 20° and 30°) on the irrigation effectiveness in a real root canal. In this paper, the distance between the tips of the root canal and the needle was adopted as the working depth. It means the larger the working depth, the farther away from the tip of root canal. The depth in this paper, such as the replacing depth in the following chapter, is defined as the distance from the tip end of the root canal. The smaller the depth is, the deeper the position in the root canal is. Efficient replacement of fluid at the apical area of the root canal, the shear stress on the canal wall and the average pressure at the apical area of the root canal were important parameters in measuring the irrigation effectiveness.

### Effect of working depth

**Fig. 2:**
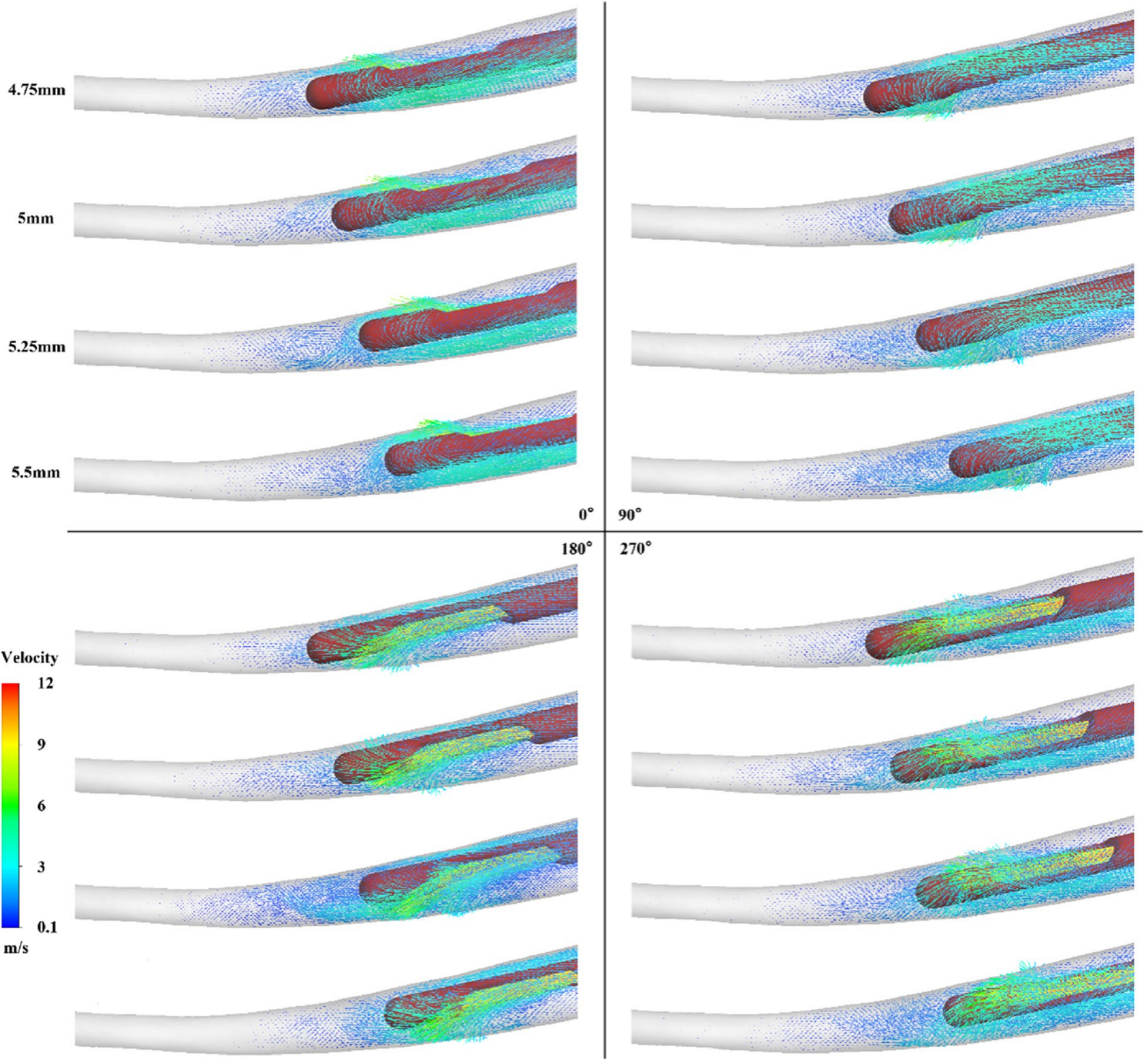
Effect of working depth and side-vented needle orientation on the irrigation flow field

Figure 2 shows the flow field pattern of the irrigation at different working depths and orientations. The different needle outlet orientations at a working depth of 4.75 mm have little influence on the flow field, and the difference in the flow of the irrigation at the apical root canal is negligible. At a working depth of 5 mm, the flow field pattern for different orientations varies. The irrigation flows at the apical root canal at 90° and 270° are significantly attenuated compared to those at the other orientation angles. Moreover, there are subtle differences in the flow field for different needle outlet orientations at a working depth of 5.25 mm, where some recirculation is apparent. Such recirculation zone helps the flow field reaches a greater depth in the root canal. In the irrigation flow field at a working depth of 5.5 mm, the top flow field at 180° is reduced. There are few obvious backflow structures and local vortices in the flow field at 90° and 270°.

**Fig. 3:**
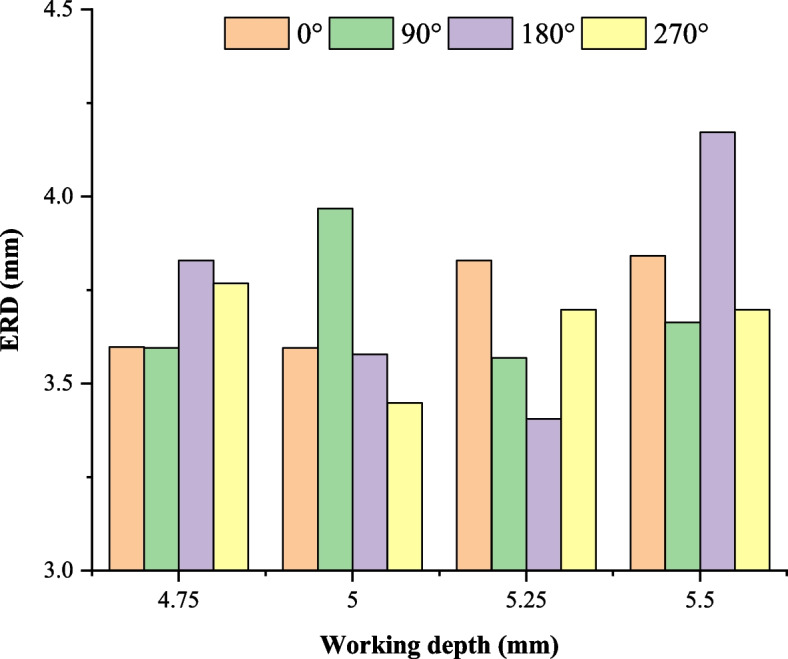
Effective replacing depth (ERD) at different needle orientations

Because of the non-uniform distribution in transverse and longitude directions of both the real root canal and the needle nozzle, a definite non-uniform circumstantial distribution of the flow field can be obtained during irrigation. As a result, the maximum depth of the irrigation field differs apparently from each other while needle orientations are different. In this paper, a velocity greater than 0.1 m/s is commonly regarded as the minimum effective velocity for flushing away the polluted fluid and replacing it with fresh irrigation [5]^.^ The minimum depth of the effective velocity in longitude direction is then regarded as the effective replacing depth (ERD). Figure 3 shows the ERD at different needle orientations. It can be found that the ERD at different orientation differs apparently from each other even when the same working depth is adopted. And no obvious regular variation of the ERD can be observed directly.

**Fig. 4:**
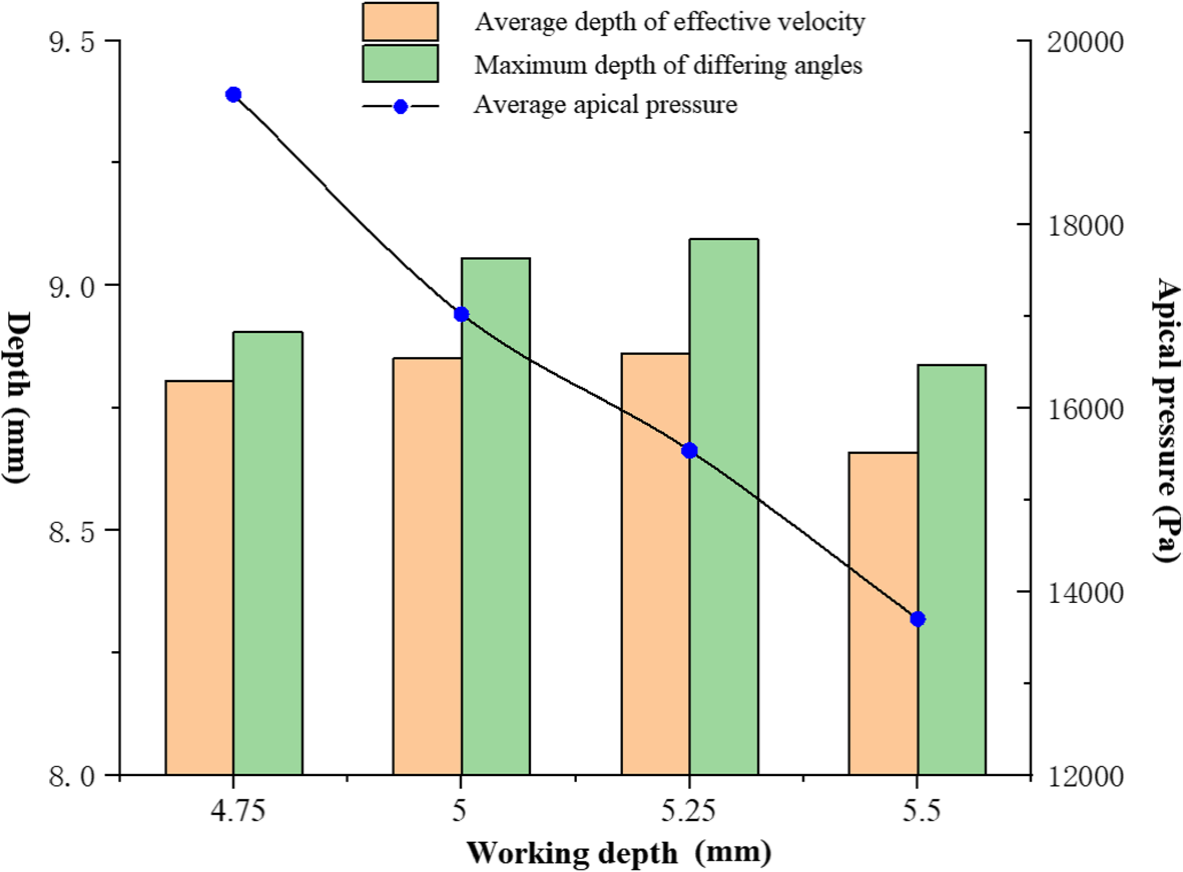
The effective replacing depth (ERD) of irrigant and apical pressure at different working depths

The overall average ERD at a working depth generally represents the coverage in depth of the irrigation. Nevertheless, the minimum ERD is another important characteristic parameter because the needle usually rotates during the irrigation process. Figure 4 compares the maximum depth to the average depth that the effective irrigation flow field reaches. First, the average ERD and the minimum ERD change similarly with the working depths. The minimum working depth (4.75 mm) that the needle reaches does not necessarily provide optimum cleaning. The highest cleaning efficiency (minimum ERD) basically appears at the working depth of 5 mm and 5.25 mm. At the working depth of 5.5 mm, cleaning efficiency decreases significantly. Figure 4 also shows the average apical pressure at different working depths. The average apical pressure decreases monotonically with the working depth. It means that irrigation with a larger working depth is safer.

**Fig. 5:**
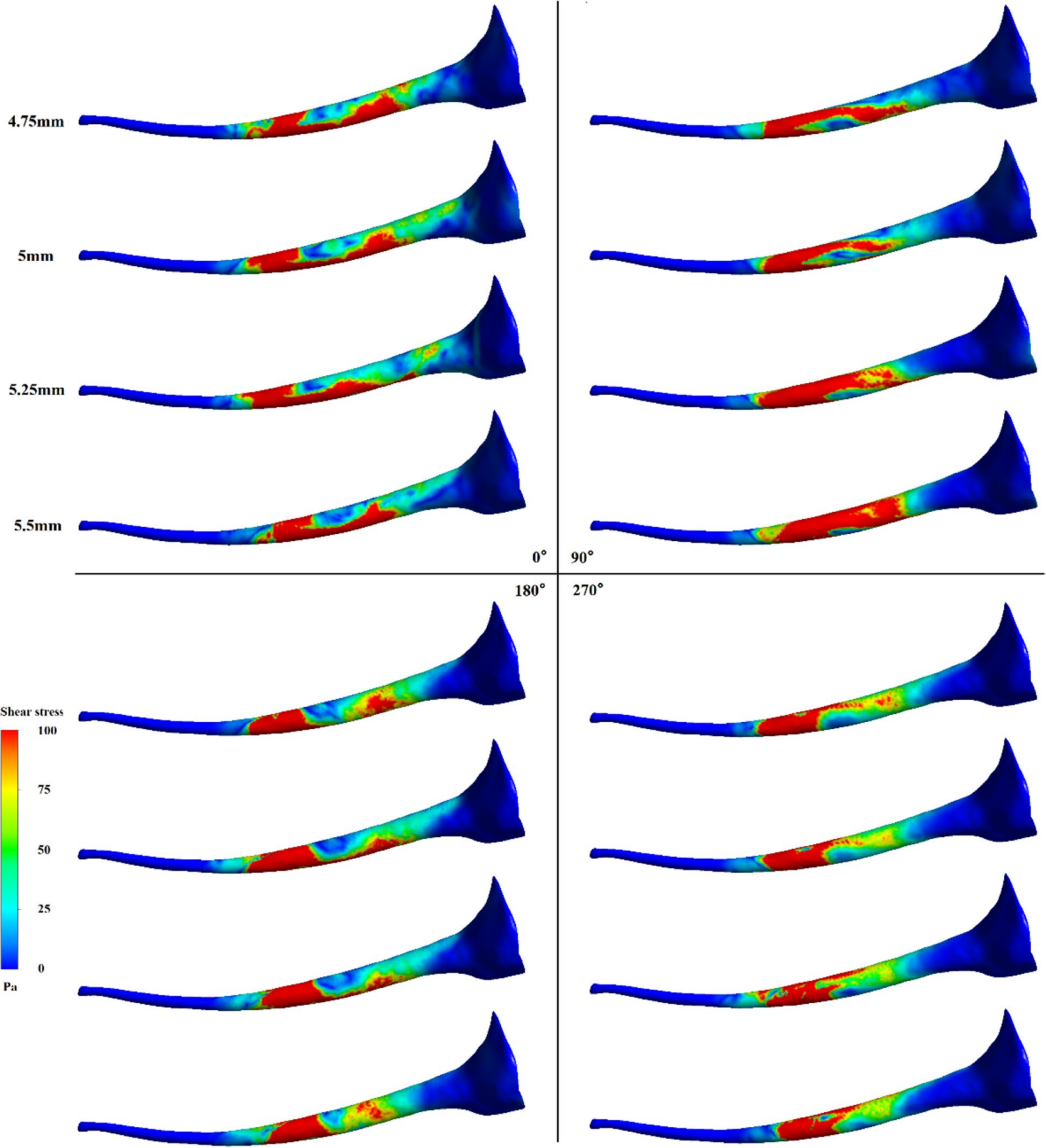
Effect of working depth and side-vented needle orientation on shear stress

During the irrigation process, the shear stress of the irrigation flow on the canal wall is key to removal of the smear layer. Wall shear stress above 100 Pa is considered to be the effective shear stress (ESS) that can remove the smear layer [7, 23]. Figure 5 shows the shear stress distribution on the root canal wall. Obviously, the distribution of wall shear stress varies widely with the needle’s working depth and outlet orientation. For example, at a working depth of 4.75 mm, the shear stress is adequate in a ring near the needle outlet, and is distributed in a long strip on the wall near the orifice of the root canal. However, as the orientation of the needle changes, the position of this strip of shear stress near the root canal orifice changes with different positions of the needle. The difference in the maximum depth that the ESS can reach does not differ greatly, but usually appears the greatest at 5 mm and 5.25 mm for the four orientations.

**Fig. 6:**
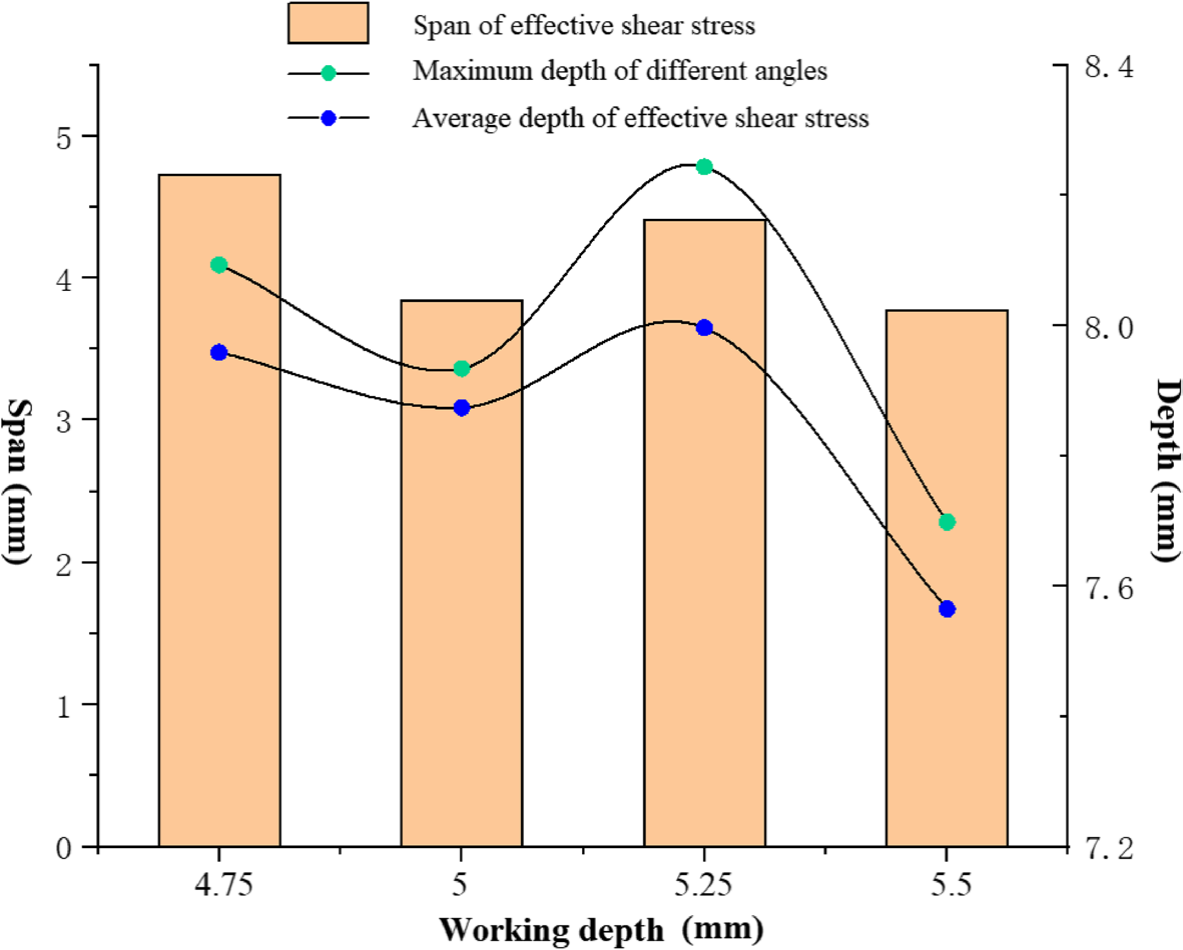
Effective Shear Stress (ESS) span and depth at different working depths and side-vented needle orientations

Span is the irrigation touch the canal wall in the transverse range. Figure 6 shows the span, the lowest depth and the average depth of ESS for different orientations. The largest span of ESS occurs when the working depth is 4.75 mm, and it comes to the minimum at the working depth of 5.5 mm. The lowest depth and the average depth of ESS appears at the working depth of 5.25 mm, indicating less smear layer is removed in irrigation. It denotes the ESS mainly distributes near the outlet of the needle when the working depth is short.

### Effect of different root canal curvatures

**Fig. 7:**
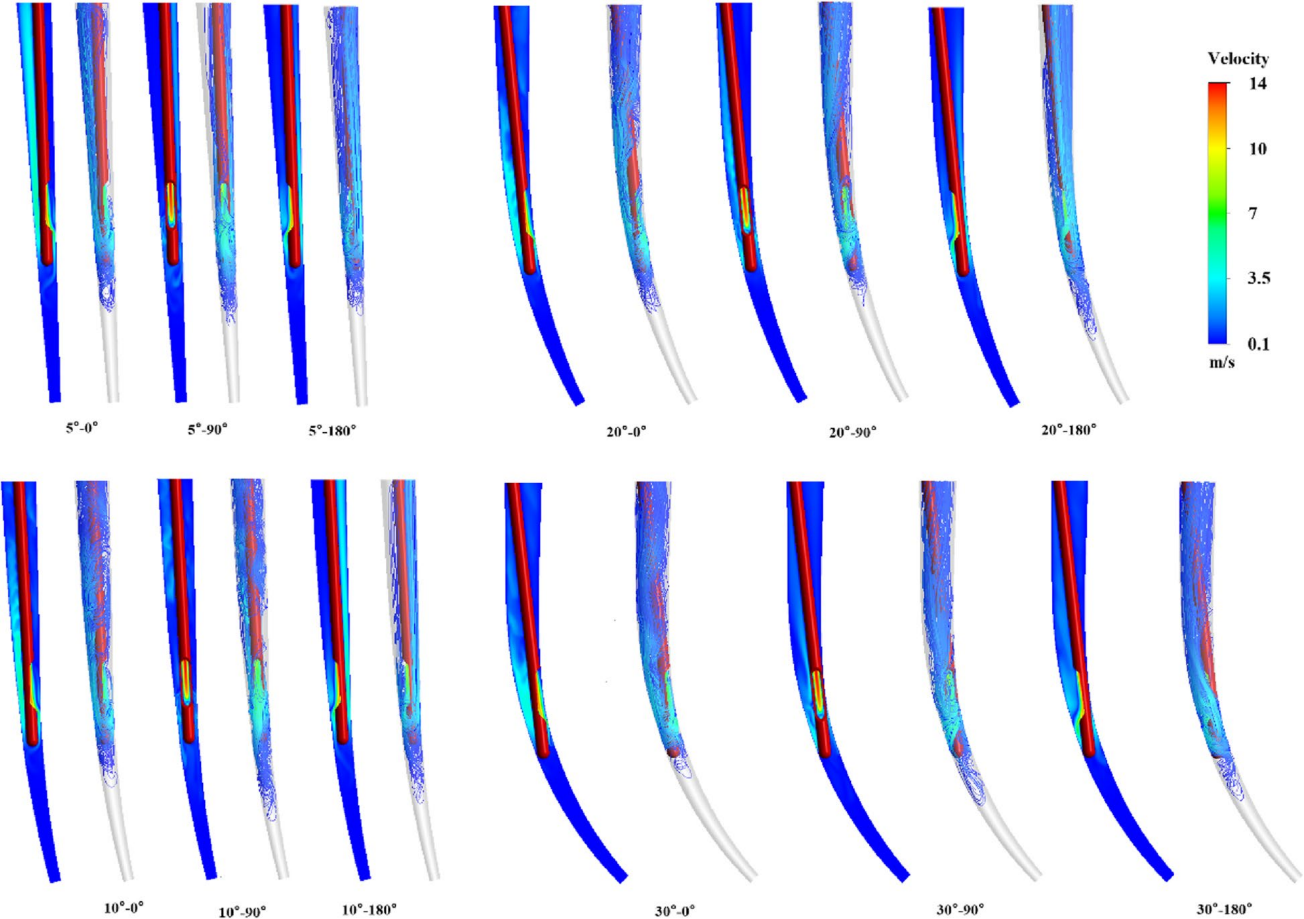
Effect of curvature and side-vented needle orientation on the irrigation flow field

Figure 7 shows the velocity flow fields (0.1 m/s-14 m/s) at different orientations of the side-vented needles with four different degrees of curvature. Orientation of the needle outlet influences the flow pattern of the irrigation during root canal irrigation. In 5° and 10° root canals, needle orientations from 0° to 90°, and the effective irrigation flow extending to the apical root canal, improve replacement of the irrigation fluid. Nevertheless, with the needle changed by a 90° to 180°, the ERD of irrigation fluid toward the apex of the root canal significantly rises. However, the effect in the 20° root canal is opposite. The ERD of the irrigation at 0° and 180° is deeper, but at 90° is shallower.

**Fig. 8:**
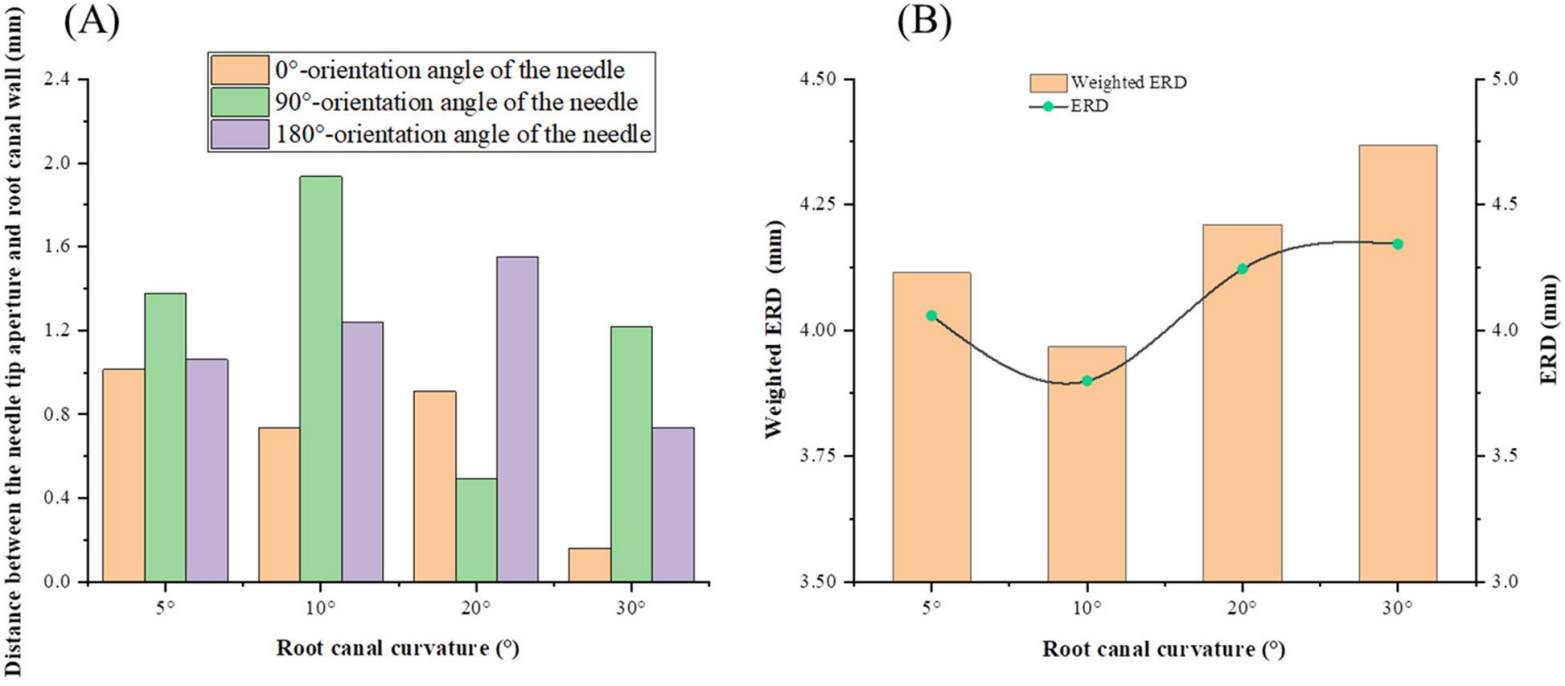
The effective replacing depth (ERD) of irrigant for different curvatures and side-vented needle orientations, (**A**) the distance between needle tip aperture and root canal wall, (**B**) ERD and weighted ERD

Figure 8A also illustrates the influence of different curvatures on the flow in root canal irrigation. Due to the limited space between the needle aperture and the 30° root canal wall, the flow range of the 30° irrigation is limited to the area near the aperture of needle tip. In this group, the replacement capacity of the irrigation for the 10° root canal is best. When the curvature increases to 20° or decreases to 5°, the depth that the effective velocity of the irrigation attains is significantly reduced. Figure 8B shows the ERD for different curvatures at different orientation angles of the needle and the circumstantially averaged ERD. The orientation angle of 90° and 270° are equivalent because of the symmetry of the root canal so that the ERD at the angle of 270 is not designated in the figure. No apparent variation trends can be observed for different orientation angles when the root canal curvature varies. Considering the needle orientation can be modified during irrigation, the weighted circumstantially averaged ERD is adopted to characterize the replacing capability. As found in Fig. 8B, the ERD reaches the lowest in to the root canal at the curvature of 10° and then increases with the curvature discrepancy.

**Fig. 9:**
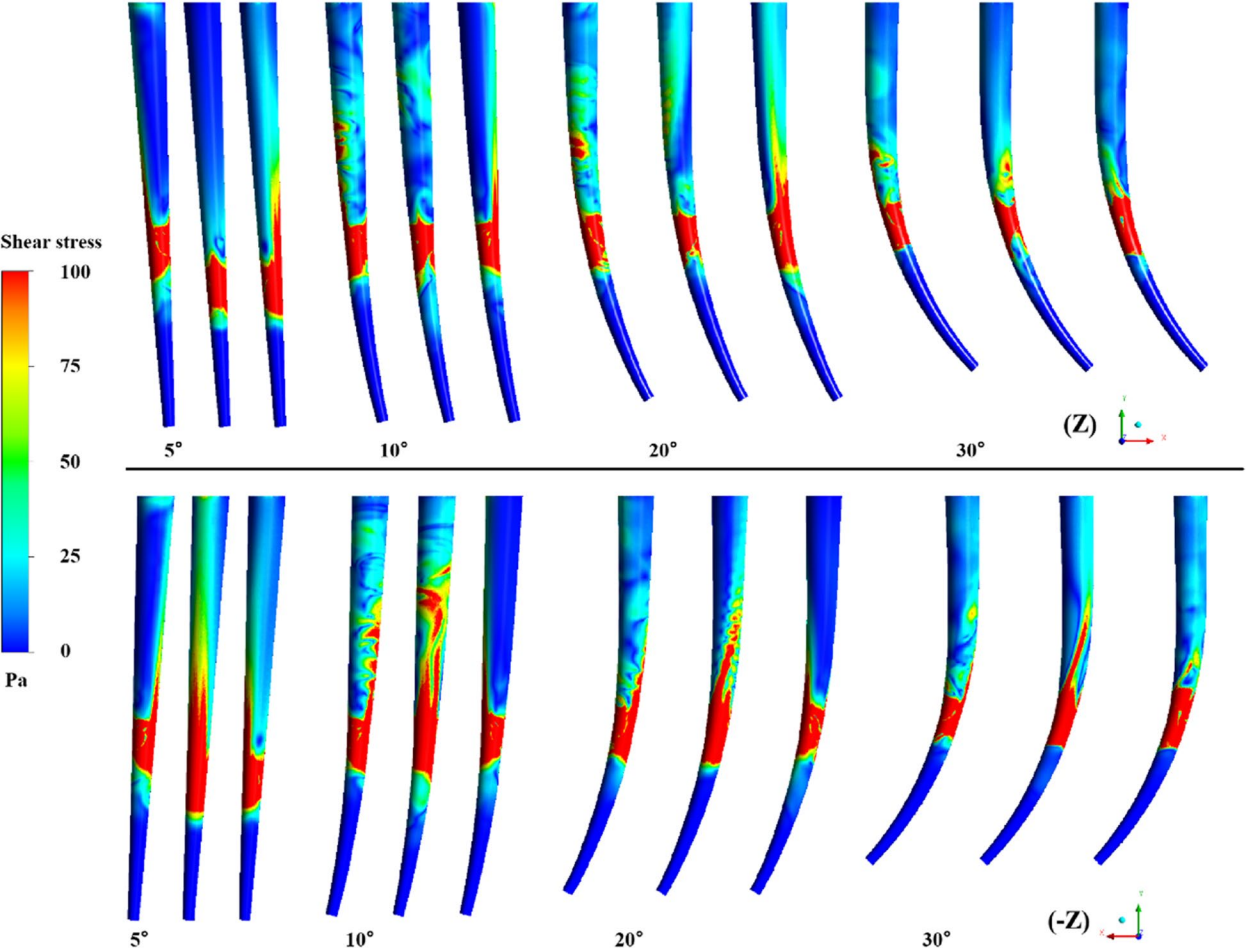
Effect of curvatures and side-vented needle orientation on shear stress

Figure 9 shows the effect of root canal curvature on the wall shear stress distribution. The red area represents the coverage of ESS on the wall. It can be observed in both front and rear directions that ESS mainly appears on the wall around the needle outlet. Apparently different distribution of ESS, including the depth it reaches, the circumstantial and radial distribution, is observed not only between different curvatures, but also between different orientations of the needle aperture even when the root canal curvature is the same.

**Fig. 10:**
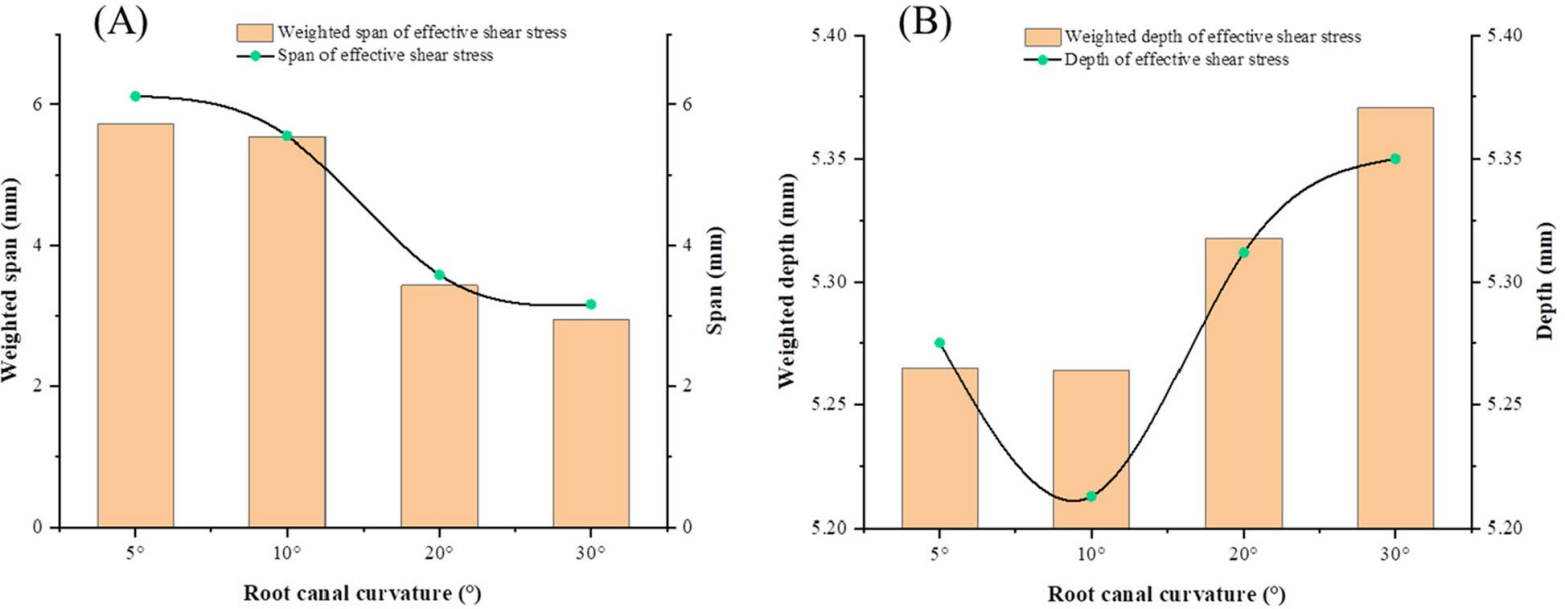
Effective Shear Stress (ESS) span and depth in different curvatures, (**A**) ESS span, (**B**) ESS depth

Figure 10 shows the radial span and reaching depths of ESS for different root canal curvatures. Considering the apparently different distribution of different needle orientations and the rotation of the needle during irrigation, both the maximum value and the averaged value of all orientations are then obtained for the span (as shown in Fig. 10a) and depth (as shown in Fig. 10b), respectively. The maximum span and the average span simultaneously vary with the curvature. Basically, the ESS span decreases with the curvature, especially when the curvature is > 10°. The similar variation trend of the maximum and average ESS depths is also obtained. The minimum value, which denote the t lowest amount of irrigation, occurs at the curvature of 10°, the value of depth is obviously much larger when the curvature is > 20°, indicating that larger root canal curvature hiders the irrigation efficiency in transverse direction but accelerates it in and longitude direction with a side-vented needle.

## Discussion

Some studies based on CFD have been published in the field of root canal irrigation, but few discuss simulated root canal derived from natural tooth. CFD simulation can obtain the irrigation flow pattern, shear stress and apical pressure, which effectively influence the irrigation efficiency. This study makes use of micro-CT and three-dimensional reconstruction technology to acquire a realistic root canal model after the initial preparation. In clinical treatment and scientific research, the 30-G side-vented irrigation needle is commonly used [24]. Unlike another widely used flat needle, the coupling of side vent and the curvature of the real root canal can effectively modify the irrigation flow field and thereby influence the final efficiency of the irrigation. To investigate the irrigation effect of the side-vented needle in real root canals with different curvatures, the outlet of the side-vented needle was designed for four orientations because of the definite difference of the flow field between the four orientations.

In present study, the irrigation process at four working depths and four different curvatures were investigated to analyze the irrigation effect of the side-vented needle in real root canals. In previous studies, the irrigating effect of the side-vented needle has been analyzed for various factors, such as the orientation angle of the needle and the working depth [11, 13], and the effect of irrigation in root canals of different curvatures was studied in the laboratory [25]. However, on the effect of the root canal curvatures and needle positions on the irrigation with side-vented needle has been carried out in mature first molar root canals. And also because of the anisotropic distribution of the opening of the needles, the influence of the orientation of the side-vented needle outlet is considered. The uniqueness of real root canals determines the results and interpretations of the cases in this study to be important in actual occasions.

It was disclosed that improving the working depth of a flat needle didn’t necessarily improve the irrigation efficiency in our former research [20]. The irrigation effect of the side-vented needle is also significantly affected by the working depth through the irrigation simulation of the side needle at four working depths. Meanwhile, the difference in the effect of working depths is closely related to the orientation of the needle outlet. At a working depth of 5 mm, ERD in the 90° orientation is larger than those of the other three orientations. At the 90° orientation and working depth of 5 mm, the irrigation directly impacts the wall of the root canal after flowing out of the needle, and great energy dissipation at the impact point impedes to the penetration of the irrigation flow to the apex. The deepest penetration (the smallest ERD) of the irrigation at 180° occurs at the work depth of 5.25 mm, while the deepest penetration at the needle orientation angle of 270° is 5 mm. No obvious variation trend of ERD can be obtained with the working depth, because of the collaboration of the curving real root canal and the needle orientation. Neither the largest nor the lest working depth obtains the minimum ERD. It denotes an appropriate working depth helps to obtain the optimal replacing efficiency. Nevertheless, the average apical pressure reduces apparently with the working depth, which reduces risk of apical extrusion during actual irrigation. Therefore, the irrigation at the appropriate working depth should be selected taking account of both the displacement efficiency and the safety of apical extrusion.

The magnitude of the shear stress of the irrigation flow near the wall stands for the capability to remove the residual biofilm and debris on the wall in root canal irrigation. Wall shear stress of 100 Pa is adopted to be the minimum value that can remove the smear layer in root canal irrigation [7, 20]. The shear stress distribution shows that even the wall at the back of the side-vented needle can be completely cleaned near the outlet. Definite difference of the maximum depth and the axial span that the ESS on the wall can reach at different needle orientations can be observed. Nevertheless, the circumstantial average depth, the maximum depth and the axial span of the ESS don’t vary synchronously with the working depth. Thus, the average depth, the maximum depth and the axial span of the ESS are all adopted to assess the wall cleaning efficiency during irrigation. Working depth clearly influences the ESS depths and distribution. Basically, the working depths of 4.75 mm and 5.25 mm have the relatively higher efficiency in wall cleaning. It means the irrigation conditions for the best irrigation replacement doesn’t necessarily lead to the best removal and ablution of the smear layer in root canal. This finding differs from the previous results for simple tapered root canals at different depths [26]. Meanwhile, the curvature of the root canal can also severely influence the clean depth of the wall. It can be found both the span and the depth of the ESS for little curvatures (5° and 10°) root canals are higher than those for large curvatures (20° and 30°). But for little curvatures, no apparent difference between 5° and 10° can be observed. It also denotes the large curvature of the root canal can apparently improve the difficulty in irrigation. Due to the collaboration of the side-opened outlet and the curvature of the root canal, it can be also observed the smallest depth occurs at the curvature of 10°. It implies certain advantages of side-vented needles in removing the smear layer in the irrigation of a curving root canal.

There are laboratory studies on the irrigation effect of real root canal curvature [14], but no useful conclusions have been drawn, and a CFD method able to acquire the internal parameters of the irrigation flow field has not been applied to research into curvature. CFD study of the simple conical root canals finds dead water at the apical root canal, where irrigation does not flow during irrigation. This hinders the replacement of irrigation and the removal of the smear layer [15, 21]. In present study, the smallest dead water zone seems to occur when the curvature is 10°. It may result from the collaboration of the curvature and the side-opened outlet.

## Conclusions

In real root canals, due to the influence of the curvature of the root canal, the irrigation flow fields appear to differ apparently between different needle orientation, which makes the replacement efficiency varies with the curvature and the working depth. Correspondingly, the shear stress of the irrigation flow near the wall change significantly, which severely influences the removal efficiency of the smear layers of the root canal. In this study, the model of the real root canal of the first maxillary molar having been prepared, the effect of the side-vented needle at different working depths and the root canal curvatures on the irrigation is then discussed, in which the outlet orientation of the side-vented needle (0°, 90°, 270°) was considered.

1. With increase in working depth, the ERD of the irrigation flow is generally improved at the working depths of 4.75 mm and 5.25 mm, implying the moderate working depth helps to improve replacement capacity. The apical pressure decreases with the working depth increase, which denotes the lower risk of apical extrusion.
2. The depth of the stresses doesn’t vary synchronously with the ERD at different working depth. It denotes the irrigation conditions for the best irrigation replacement doesn’t necessarily lead to the best removal and ablution of the smear layer in root canal.
3. The curvature of the root canal can severely influence the removal depth of the smear layer on the wall. It can be found both the span and the depth of the ESS for little curvatures (5° and 10°) root canals are higher than those for large curvatures (20° and 30°). But for little curvatures, no apparent difference between 5° and 10° can be observed. It also denotes the large curvature of the root canal can apparently improve the difficulty in irrigation.
4. The smallest dead water zone in front of the needle occurs when the curvature of the root canal is 10°, resulting from the collaboration of the curvature and the side-vented needle. Meanwhile, the smallest depth of ESS near the wall also appears at the curvature of 10°. These results denote the advantages of side-vented needles in improving the irrigation efficiency of a curving root canal.

## Data Availability

The datasets generated during and analyzed during the current study are not publicly available due to their containing information that could compromise the privacy of research participant, but are available from the corresponding author on reasonable request.

## Abbreviations

Micro-CT: Micro-Computed Tomography
CFD: Computational fluid dynamics
D_ext_: External Diameter
D_int_: Internal Diameter
L: Length
