# Correction to: Computational fluid dynamics investigation on the irrigation of a real root canal with a side-vented needle

**DOI:** 10.1186/s12903-024-04276-9

**Published:** 2024-05-07

**Authors:** Mingzhou Yu, Yi Li, Mengdie Zhao, Zhengqiu Huang, Na Zhou, Hanhui Jin

**Affiliations:** 1 School of Aeronautics and Astronautics, Zhejiang University, Hangzhou, 310027 China; 2 Aerosol Science and Technology Laboratory, China Jiliang University, Hangzhou, 310018 China; 3 Stomatology Hospital, School of Stomatology, Zhejiang University School of Medicine, Clinical Research Center for Oral Diseases of Zhejiang Province, Key Laboratory of Oral Biomedical Research of Zhejiang Province, Cancer Center of Zhejiang University, Hangzhou, 310006 China; 4 State Key Laboratory of Clean Energy Utilization, Zhejiang University, Hangzhou, 310027 China

**Correction to: BMC Oral Health (2024) 24:321**

10.1186/s12903-024-03966-8

In this article [1], the affiliation details for the corresponding author Na Zhou were incorrectly given as ‘2 Aerosol Science and Technology Laboratory, China Jiliang University, Hangzhou 310018, People’s Republic of China and 3 Stomatology Hospital, School of Stomatology, Zhejiang University School of Medicine, Clinical Research Center for Oral Diseases of Zhejiang Province, Key Laboratory of Oral Biomedical Research of Zhejiang Province, Cancer Center of Zhejiang University, Hangzhou 310006, People’s Republic of China.’ but should have been only 3 Stomatology Hospital, School of Stomatology, Zhejiang University School of Medicine, Clinical Research Center for Oral Diseases of Zhejiang Province, Key Laboratory of Oral Biomedical Research of Zhejiang Province, Cancer Center of Zhejiang University, Hangzhou 310006, People’s Republic of China.
